# Blockade of endothelial Mas receptor restores the vasomotor response to phenylephrine in human resistance arterioles pretreated with captopril and exposed to propofol

**DOI:** 10.1186/s12871-022-01786-3

**Published:** 2022-07-29

**Authors:** Mary E. Schulz, Joseph C. Hockenberry, Boran Katunaric, Paul S. Pagel, Julie K. Freed

**Affiliations:** 1 Department of Anesthesiology, Medical College of Wisconsin, 8701 Watertown Plank Road, Milwaukee, WI 53226 USA; 2 Cardiovascular Center, Medical College of Wisconsin, Milwaukee, WI 53226 USA; 3 Department of Medicine, Medical College of Wisconsin, Milwaukee, WI 53226 USA; 4 Anesthesiology Service, Clement J. Zablocki Veterans Affairs Medical Center, Milwaukee, WI 53295 USA; 5 Department of Physiology, Medical College of Wisconsin, Milwaukee, WI 53226 USA

**Keywords:** Angiotensin converting enzyme inhibitor, Ang 1–7, Mas receptor, Propofol, Vasodilation, Resistance arterioles

## Abstract

**Background:**

Hypotension that is resistant to phenylephrine is a complication that occurs in anesthetized patients treated with angiotensin converting enzyme (ACE) inhibitors. We tested the hypothesis that Ang 1–7 and the endothelial Mas receptor contribute to vasodilation produced by propofol in the presence of captopril.

**Methods:**

The internal diameters of human adipose resistance arterioles were measured before and after administration of phenylephrine (10^–9^ to 10^–5^ M) in the presence and absence of propofol (10^–6^ M; added 10 min before the phenylephrine) or the Mas receptor antagonist A779 (10^–5^ M; added 30 min before phenylephrine) in separate experimental groups. Additional groups of arterioles were incubated for 16 to 20 h with captopril (10^–2^ M) or Ang 1–7 (10^–9^ M) before experimentation with phenylephrine, propofol, and A779.

**Results:**

Propofol blunted phenylephrine-induced vasoconstriction in normal vessels. Captopril pretreatment alone did not affect vasoconstriction, but the addition of propofol markedly attenuated the vasomotor response to phenylephrine. A779 alone did not affect vasoconstriction in normal vessels, but it restored vasoreactivity in arterioles pretreated with captopril and exposed to propofol. Ang 1–7 reduced the vasoconstriction in response to phenylephrine. Addition of propofol to Ang 1–7-pretreated vessels further depressed phenylephrine-induced vasoconstriction to an equivalent degree as the combination of captopril and propofol, but A779 partially reversed this effect.

**Conclusions:**

Mas receptor activation by Ang 1–7 contributes to phenylephrine-resistant vasodilation in resistance arterioles pretreated with captopril and exposed to propofol. These data suggest an alternative mechanism by which refractory hypotension may occur in anesthetized patients treated with ACE inhibitors.

Angiotensin converting enzyme (ACE) inhibitors are commonly used for treatment of hypertension and heart failure with reduced ejection fraction (HFrEF) [1]. These medications also exert beneficial effects in patients with acute myocardial infarction and substantially reduce the incidence of major adverse cardiovascular events in patients with vascular disease or diabetes [2]. Hypotension that is resistant to treatment with phenylephrine is a well-known complication that occurs in patients who continue ACE inhibitors before undergoing general anesthesia [3, 4]. The mechanisms responsible for this phenomenon are incompletely understood. In the presence of chronic ACE inhibition, angiotensin I may be converted to the heptapeptide angiotensin 1–7 (Ang 1–7) by an alternate metabolic pathway involving neutral or prolyl-endopeptidases [5]. In contrast to the intense vasoconstriction produced by angiotensin II, Ang 1–7 activates the endothelial Mas receptor to cause vasodilation [6]. We tested the hypothesis that Ang 1–7 and the Mas receptor play important roles in vasodilation produced by propofol in the presence of ACE inhibition and further, that Mas receptor blockade with a selective antagonist restores the vasomotor response to phenylephrine by attenuating the vasodilatory actions of Ang 1–7 in human resistance arterioles.

## Materials and methods

All methods were carried out in accordance with relevant guidelines and regulations. All experimental protocols were approved by the Medical College of Wisconsin.

### Tissue acquisition

All protocols were approved by the Institutional Review Board of the Medical College of Wisconsin (MCW). Otherwise discarded fresh human adipose tissue (subcutaneous, visceral, peritoneal) was collected from patients undergoing various surgical procedures. Tissues were immediately placed in ice-cold HEPES buffer. De-identified patient demographic data was obtained using REDCap, a secure MCW clinical database.

### Vasoconstriction measurements using videomicroscopy

**Fig. 1:**
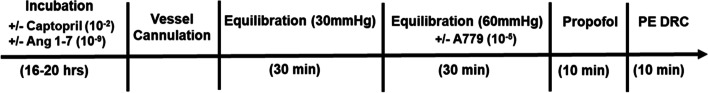
Outline of experimental protocol. Following incubation for 16-20 h with Captopril (10^–2^), Ang 1–7 (10^–9^), or vehicle, human microvessels were cannulated followed by two equilibration periods (30 mmHg and 60 mmHg, respectively). The Mas receptor antagonist A779 (10^–5^) was added during the second equilibration period. Following equilibration, vessels were exposed to propofol for 10 min prior to a phenylephrine (PE) dose–response curve (DRC) to assess vascular reactivity

Human resistance arterioles (diameter of 100–200 µm) were dissected from fresh adipose tissue, cannulated with glass micropipettes, and placed in an organ chamber circulated with warm Kreb’s buffer as previously described [7]. Following equilibration (30 min intervals at 30 and 60 mmHg), internal vessel diameters were measured under steady-state conditions before and after administration of phenylephrine (10^–9^ to 10^–5^ M) in the presence and absence of propofol (10^–6^ M; added to the organ bath 10 min before phenylephrine) or the Mas receptor antagonist A779 (10^–5^ M; added to the organ bath 30 min before phenylephrine) in separate experimental groups. Additional groups of arterioles were incubated for 16 to 20 h with captopril (10^–2^ M) or Ang 1–7 (10^–9^ M) before experimentation with phenylephrine, propofol, and A779. An endothelium-independent vasodilator (papaverine; 10^–4^ M) was added to the organ bath at the end of each experiment to verify the integrity of the vascular smooth muscle. An outline of this experimental protocol is illustrated in Fig. 1. In vessels pretreated with captopril, Ang 1–7, or propofol, potassium chloride (72.5 mM) was added to the organ bath to assess constriction and vessel viability. Percent vasoconstriction was calculated as the (initial internal diameter – diameter at each dose of phenylephrine)/initial internal diameter × 100).

### Materials

Propofol was obtained from Fresenius Kabi USA LLC (Lake Zurich, IL). All other chemicals were purchased from Sigma-Aldrich and prepared in distilled water. Vehicle controls showed that the final concentration of distilled water had no effect on the tone or function of arterioles (data not shown).

### Statistical analysis

Data are expressed as mean ± standard error of the mean. Two-way analysis of variance (2-way ANOVA) was used to compare percent constriction between interventions. Responses at individual concentrations were compared using Tukey’s multiple comparison test. All analyses were performed using Prism version 9.0.0 (GraphPad Software, San Diego, CA). The null hypothesis was rejected when the probability value was less than 0.05.

## Results

**Fig. 2:**
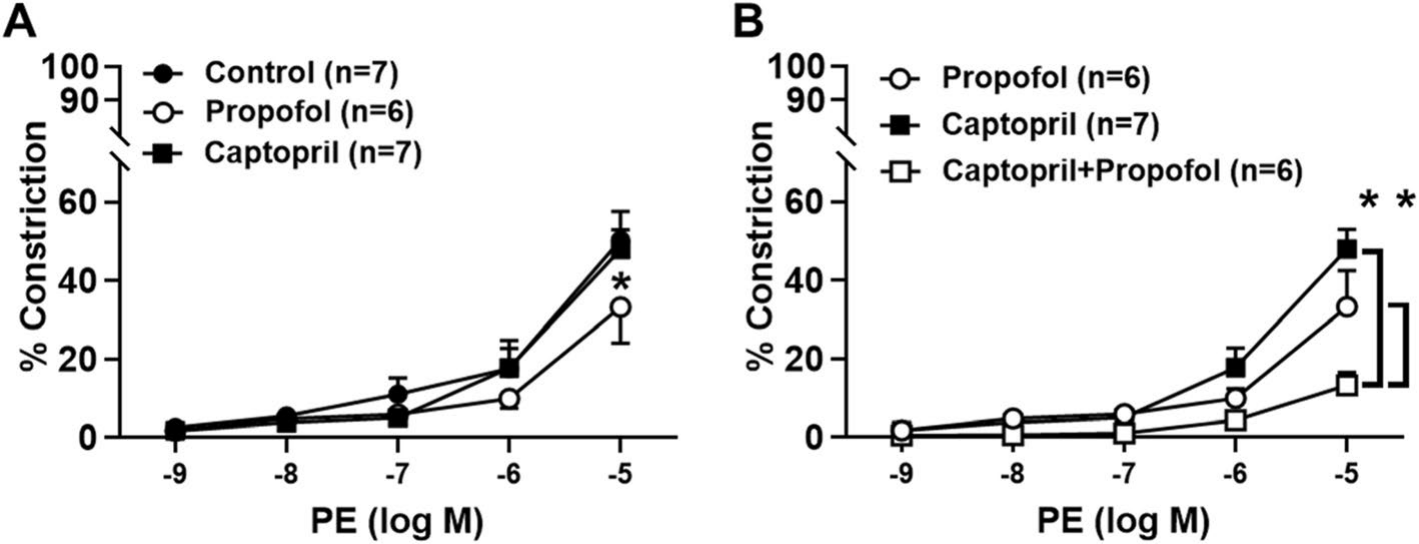
Efficacy of phenylephrine (PE) as a vasoconstrictor in human resistance arterioles. A significant (*p* < 0.05) reduction in PE-induced constriction was observed at 10^–5^ M in vessels treated with propofol alone (10^–6^ M; panel **A**). Pretreatment with captopril alone (10^–2^ M; panel **B**) did not affect alter the response to PE, but PE-induced vasoconstriction was significantly reduced in vessels treated with both captopril and propofol

Adipose tissue was obtained from 35 patients [age 48 ± 14 years (mean ± standard deviation)], 20 of whom were women. Thirty-one patients were healthy, whereas three had hypertension and one was an active tobacco abuser. Propofol blunted phenylephrine-induced vasoconstriction in normal vessels (maximum constriction at 10^–5^ M of 33% ± 9% versus 50% ± 7%; Fig. 2A). Captopril pretreatment alone did not affect vasoconstriction compared to control (48% ± 5.0, Fig. 2A), but the addition of propofol markedly attenuated phenylephrine-induced constriction (13% ± 3%; Fig. 2B).

**Fig. 3:**
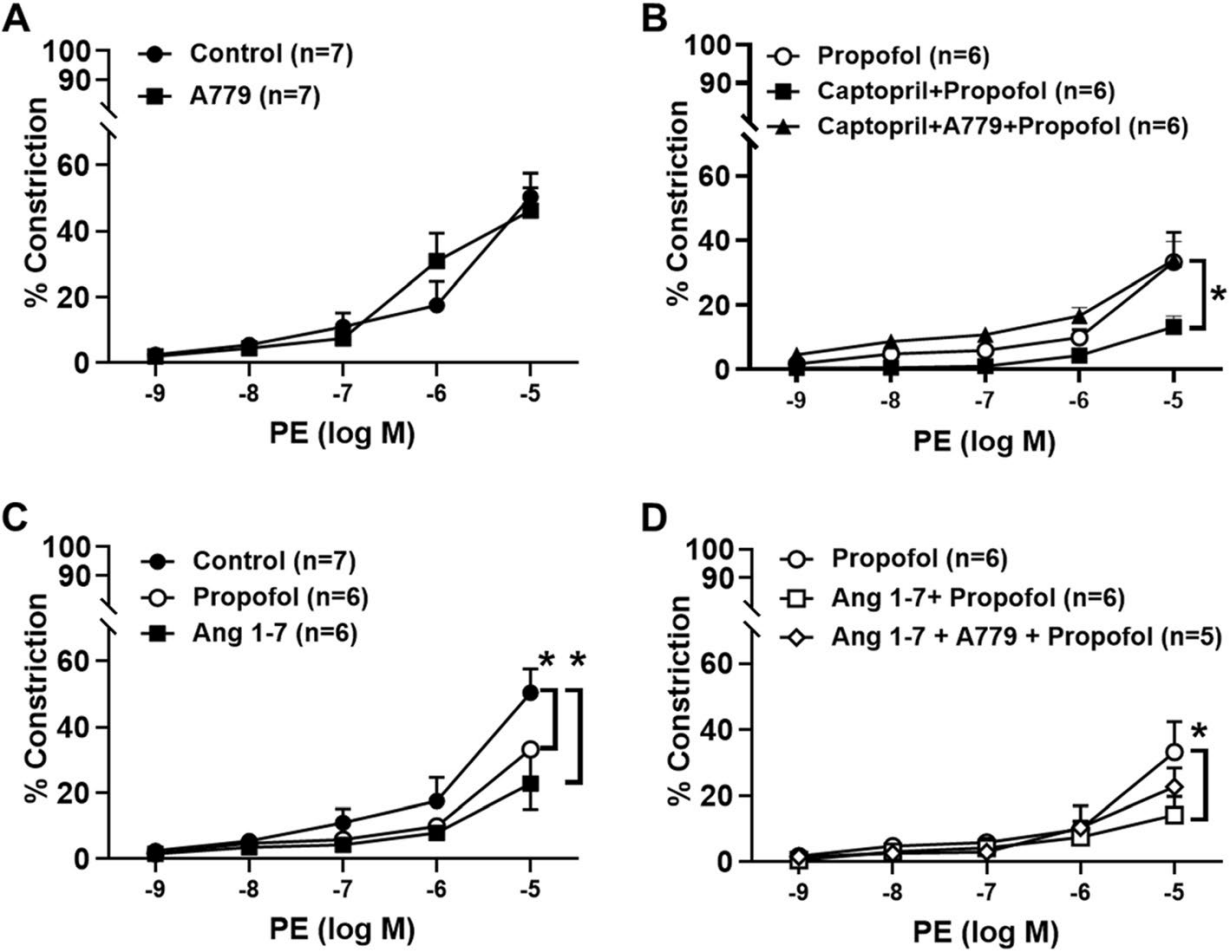
Role of the Mas receptor in phenylephrine resistance in captopril pretreated arterioles exposed to propofol. The Mas receptor antagonist A779 (10^–5^ M) alone did not affect PE-induced vasoconstriction versus control (panel **A**). Addition of A779 before propofol restored the ability of PE to constrict arterioles pretreated with captopril (panel **B**). Ang 1–7 alone (10^–9^ M) did not affect PE-induced constriction (panel **C**). PE-induced vasoconstriction of arterioles pretreated with Ang 1–7 was significantly (*P* < 0.05) attenuated compared with those exposed to propofol alone (panel **D**). However, A779 partially restored the vasoconstrictive effect of PE in microvessels pretreated with Ang 1–7 during propofol exposure to propofol

The Mas receptor antagonist A779 alone did not affect phenylephrine-induced vasoconstriction in normal vessels (Fig. 3A), but A779 restored vasoreactivity in resistance arterioles pretreated with captopril and exposed to propofol (34% ± 6%; Fig. 3B) compared to captopril and propofol alone (13.2% ± 3.3%, Fig. 3B). Incubation with Ang 1–7 reduced the vasoconstriction in response to phenylephrine (23% ± 8%; Fig. 3C). Addition of propofol to Ang 1–7-pretreated vessels further depressed phenylephrine-induced vasoconstriction (14% ± 6%; Fig. 3D) to an equivalent degree as the combination of captopril and propofol. Vessels treated with A779 following Ang 1–7 and propofol treatment appeared to have improved capacity to vasoconstrict however was not significantly different to Ang 1–7 and propofol alone (23% ± 6%; Fig. 3D).

## Discussion

**Fig. 4:**
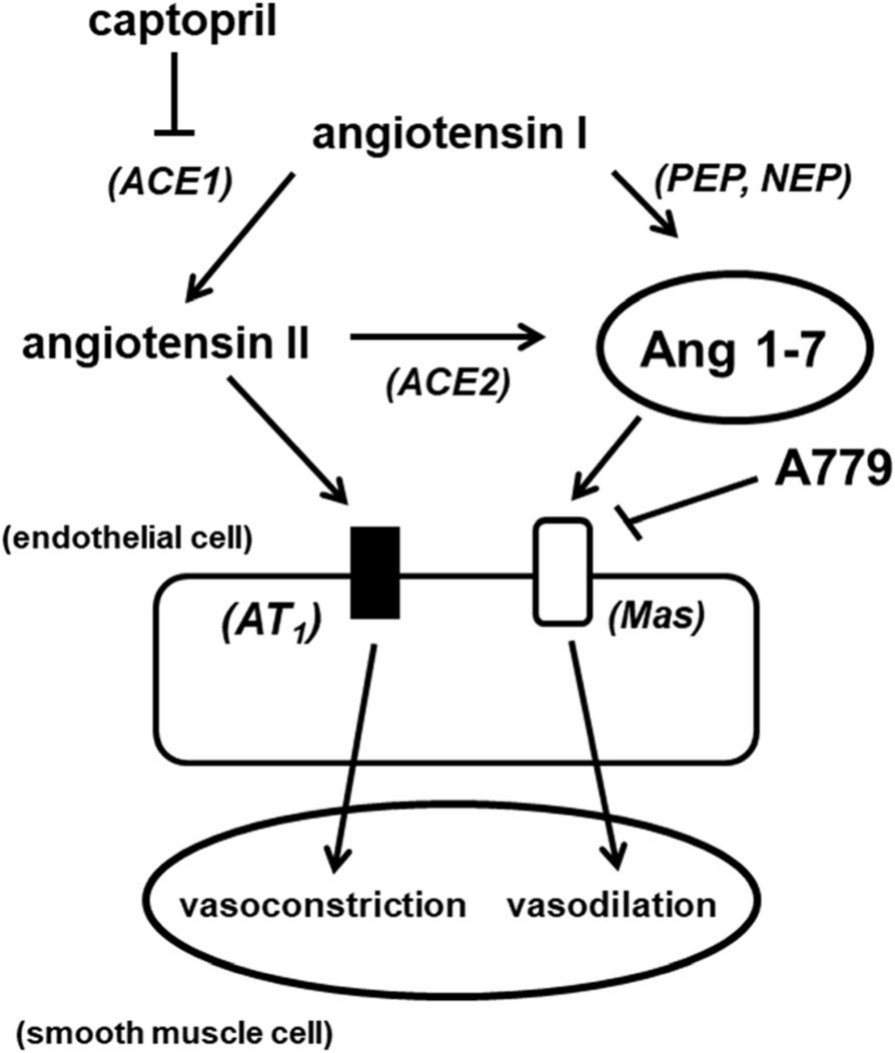
Schematic illustration representing alternative metabolism of angiotensin I and the role of the Mas receptor in attenuation of phenylephrine-induced vasoconstriction during ACE inhibition in the presence of propofol. Abbreviations: ACE1 = angiotensin converting enzyme 1; ACE2 = angiotensin converting enzyme 2; PEP = prolyl-endopeptidase; NEP = neutral endopeptidase; AT1 = angiotensin receptor 1

Phenylephrine-resistant hypotension has been extensively described in anesthetized patients when ACE inhibitors are continued during the perioperative period [8]. Decreases in circulating angiotensin II concentration due to chronic ACE inhibition and reduced sensitivity of smooth muscle cells to vasoactive medications including phenylephrine in the presence of vasodilating anesthetics such as propofol are proposed mechanisms for this hypotensive effect. The current results suggest a third possible cause for this phenomenon. Angiotensin II acts on angiotensin II type 1 and 2 (AT1 and AT2, respectively) receptors to cause arterial vasoconstriction, aldosterone secretion, and vasopressin release. When ACE is inhibited, angiotensin II is not formed and the angiotensin I that accumulates may be shunted through an alternative metabolic pathway involving neutral or prolyl-endopeptidases to form Ang 1–7 (Fig. 4).

Circulating angiotensin II may also be converted to Ang 1–7 through a second form of ACE that is not blocked by clinically used ACE inhibitors, a process that is more likely to occur in the presence of an angiotensin receptor blocker [9]. Ang 1–7 is capable of binding to AT1 and AT2 receptors [10], but it has a much higher affinity (K_D_ = 0.83 nmol/L) for the Mas receptor [11]. Unlike the vasoconstrictor angiotensin II, Ang 1–7 is a potent vasodilator that acts through the phosphoinositol-3-kinase pathway to generate nitric oxide by stimulating endothelial nitric oxide synthase [12]. As a result of these observations, we speculated that Ang 1–7 and the Mas receptor contribute to phenylephrine resistance during ACE inhibition and exposure to propofol.

Our results indicate that incubation of resistance arterioles with captopril alone did not impair vasoconstriction to phenylephrine, but the addition of propofol to the vessel bath markedly attenuated the phenylephrine-induced vasoconstrictor response. These results in an isolated vessel model mimic the observations encountered clinically in some anesthetized patients treated with ACE inhibitors in whom hypotension resulting from vasodilation is unresponsive to administration of phenylephrine. Our findings also indicate that blockade of the Mas receptor with the selective antagonist A779 restores phenylephrine-induced constriction of resistance arterioles in the presence of captopril and propofol. The results further demonstrate that administration of the Ang 1–7 mimics the vasodilation produced by ACE inhibition during exposure to propofol and that these effects are blocked by A779. Overall the vasoplegic effect elicited by exogenous Ang 1–7 compared to captopril appeared to be more robust as administration of Ang 1–7 alone induced resistance to phenylephrine-induced constriction (Fig. 3C) as opposed to captopril. It is possible that the levels Ang 1–7 produced endogenously by the shunting of angiotensin I to Ang 1–7 during ACE inhibition with captopril is much lower than the exogenous concentration of Ang 1–7 used in this study (10^–9^). Collectively, the findings suggest that increases in Ang 1–7 and Mas receptor activation contribute to vasodilation in captopril-pretreated arterioles when a clinically relevant concentration of propofol is present. The results also raise the distinct possibility that Ang 1–7 is a mediator of phenylephrine-resistant hypotension in patients chronically treated with ACE inhibitors who receive vasodilating anesthetics through its actions on the Mas receptor.

Our results should be interpreted within the constraints of several potential limitations. Mas antagonism with A779 was beneficial in restoring responsiveness to phenylephrine in Ang 1–7 treated vessels (Fig. 3D), but this effect was more robust in vessels incubated with captopril (Fig. 3B). These findings suggest that the relative concentration of Ang 1–7 generated in captopril-treated vessels may have been less than that provided exogenously (10^–9^ M). The enzyme-linked immunosorbent assay (ELISA) technique can be used to quantify Ang 1–7 concentrations, but we were unable to use ELISA for this purpose here because of the very small sample volume contained within lumen of an isolated human arteriole. Alternatively, we could have used whole intact microvessels to facilitate Ang 1–7 measurement, but such an approach would not have distinguished endothelial from vascular smooth muscle Ang 1–7. Our experiments were performed using resistance arterioles obtained from healthy subjects, most of whom did not have risk factors for heart or vascular disease, but it remains possible that these individuals may have had other conditions that affected arteriolar vasoreactivity. Whether the patients from whom adipose tissue was harvested for analysis were taking medications that affect vascular responsiveness to phenylephrine is unknown. However, thorough washing of microvessels with buffer is performed before conducting functional studies to minimize this potentially confounding effect. Most patients receive ACE inhibitors for treatment of hypertension, HFrEF, or coronary artery disease. The vasomotor responses of resistance arterioles obtained from patients with these diseases may be different than those observed here in relatively healthy subjects. Nevertheless, our observation that phenylephrine-induced vasoconstriction is markedly attenuated in captopril-pretreated microvessels from healthy individuals exposed to propofol strongly suggests that this phenomenon is a direct pharmacological effect that occurs independent of the presence of co-morbidities. A dose–response relationship to propofol was not performed, but the concentration of the intravenous anesthetic (10^–6^ M) was similar to that observed during induction of anesthesia [13]. Lastly, propofol has been previously reported to increase Ang 1–7 concentration [14], but this effect only occurs over several hours as opposed to 10-min exposure used in our experiments.

In conclusion, the current results indicate that activation of the Mas receptor by Ang 1–7 contributes to phenylephrine-resistant vasodilation in human resistance arterioles pretreated with captopril and exposed to propofol. These data suggest an alternative mechanism by which refractory hypotension may occur in anesthetized patients treated with ACE inhibitors.

## Data Availability

The datasets used/and or analyzed during the current study are available upon request. Please contact the corresponding author (J.K.F.) to request data from this study.

## References

[1] Pagel PS, Tawil JN, Boettcher BT, Izquierdo DA, Lazicki TJ, Crystal GJ, Freed JK (2021). Heart Failure With Preserved Ejection Fraction: A Comprehensive Review and Update of Diagnosis, Pathophysiology, Treatment, and Perioperative Implications. J Cardiothorac Vasc Anesth.

[2] Heart Outcomes Prevention Evaluation (HOPE) Study Investigators. Effects of ramipril on cardiovascular and microvascular outcomes in people with diabetes mellitus: results of the HOPE study and MICRO-HOPE substudy. Lancet. 2000;355:253–9. 10675071

[3] Bertrand M, Godet G, Meersschaert K, Brun L, Salcedo E, Coriat P (2001). Should the angiotensin II antagonists be discontinued before surgery?. Anesth Analg.

[4] Kheterpal S, Khodaparast O, Shanks A, O'Reilly M, Tremper KK (2008). Chronic angiotensin-converting enzyme inhibitor or angiotensin receptor blocker therapy combined with diuretic therapy is associated with increased episodes of hypotension in noncardiac surgery. J Cardiothorac Vasc Anesth.

[5] Chappell MC, Pirro NT, Sykes A, Ferrario CM (1998). Metabolism of angiotensin-(1–7) by angiotensin-converting enzyme. Hypertension.

[6] Schinzari F, Tesauro M, Veneziani A, Mores N, Di Daniele N, Cardillo C (2018). Favorable Vascular Actions of Angiotensin-(1–7) in Human Obesity. Hypertension.

[7] Freed JK, Beyer AM, LoGiudice JA, Hockenberry JC, Gutterman DD (2014). Ceramide changes the mediator of flow-induced vasodilation from nitric oxide to hydrogen peroxide in the human microcirculation. Circ Res.

[8] Liu H, Yu L, Yang LQ, Green MS (2017). Vasoplegic syndrome: An update on perioperative considerations. J Clin Anesth.

[9] Wang X, Ye Y, Gong H, Wu J, Yuan J, Wang S, Yin P, Ding Z, Kang L, Jiang Q, Zhang W, Li Y, Ge J, Zou Y (2016). The effects of different angiotensin II type 1 receptor blockers on the regulation of the ACE-AngII-AT1 and ACE2-Ang(1–7)-Mas axes in pressure overload-induced cardiac remodeling in male mice. J Mol Cell Cardiol.

[10] Galandrin S, Denis C, Boularan C, Marie J, M'Kadmi C, Pilette C, Dubroca C, Nicaise Y, Seguelas MH, N'Guyen D, Baneres JL, Pathak A, Senard JM, Gales C (2016). Cardioprotective Angiotensin-(1–7) Peptide Acts as a Natural-Biased Ligand at the Angiotensin II Type 1 Receptor. Hypertension.

[11] Savergnini SQ, Beiman M, Lautner RQ, de Paula-Carvalho V, Allahdadi K, Pessoa DC, Costa-Fraga FP, Fraga-Silva RA, Cojocaru G, Cohen Y, Bader M, de Almeida AP, Rotman G, Santos RA (2010). Vascular relaxation, antihypertensive effect, and cardioprotection of a novel peptide agonist of the MAS receptor. Hypertension.

[12] Li P, Chappell MC, Ferrario CM, Brosnihan KB (1997). Angiotensin-(1–7) augments bradykinin-induced vasodilation by competing with ACE and releasing nitric oxide. Hypertension.

[13] Fan SZ, Yu HY, Chen YL, Liu CC (1995). Propofol concentration monitoring in plasma or whole blood by gas chromatography and high-performance liquid chromatography. Anesth Analg.

[14] Zhang L, Wang J, Liang J, Feng D, Deng F, Yang Y, Lu Y, Hu Z (2018). Propofol prevents human umbilical vein endothelial cell injury from Ang II-induced apoptosis by activating the ACE2-(1–7)-Mas axis and eNOS phosphorylation. PLoS ONE.

